# New Features about Tau Function and Dysfunction

**DOI:** 10.3390/biom6020021

**Published:** 2016-04-19

**Authors:** Miguel Medina, Félix Hernández, Jesús Avila

**Affiliations:** 1 CIBERNED (Centro de Investigación Biomédica en Red de Enfermedades Neurodegenerativas), Valderrebollo 5, 28031 Madrid, Spain; mmedina@ciberned.es (M.M.); fhernandez@cbm.csic.es (F.H.); 2 CIEN Foundation, Valderrebollo 5, 28041 Madrid, Spain; 3 Centro de Biología Molecular Severo Ochoa, CSIC-UAM, Nicolás cabrera 1, 28049 Madrid, Spain

**Keywords:** Alzheimer’s disease, immunotherapy, neurodegeneration, spreading, tau, tauopathies

## Abstract

Tau is a brain microtubule-associated protein that directly binds to a microtubule and dynamically regulates its structure and function. Under pathological conditions, tau self-assembles into filamentous structures that end up forming neurofibrillary tangles. Prominent tau neurofibrillary pathology is a common feature in a number of neurodegenerative disorders, collectively referred to as tauopathies, the most common of which is Alzheimer’s disease (AD). Beyond its classical role as a microtubule-associated protein, recent advances in our understanding of tau cellular functions have revealed novel insights into their important role during pathogenesis and provided potential novel therapeutic targets. Regulation of tau behavior and function under physiological and pathological conditions is mainly achieved through post-translational modifications, including phosphorylation, glycosylation, acetylation, and truncation, among others, indicating the complexity and variability of factors influencing regulation of tau toxicity, all of which have significant implications for the development of novel therapeutic approaches in various neurodegenerative disorders. A more comprehensive understanding of the molecular mechanisms regulating tau function and dysfunction will provide us with a better outline of tau cellular networking and, hopefully, offer new clues for designing more efficient approaches to tackle tauopathies in the near future.

## 1. Introduction

From an analytical point of view, the three main cell compartments—nucleus, cytoplasm, and membrane—have been mostly studied in the past century by three different scientific disciplines: molecular biology (nucleus), cell biology (cytoplasm), and cell signaling (membrane). During the 1970s and 1980s, in an effort to introduce the molecular biology to the study of the cell cytoplasm, proteins located at the cytoplasm were analyzed. These were mainly those involved in the components of the cytoskeleton: microtubules, microfilaments, and intermediate filaments. Microtubules are highly enriched in the cytoplasm of neuronal cells and, therefore, neuronal microtubules were the first ones to be characterized at the molecular level [1].

Brain microtubules can be isolated *in vitro* and they are composed mostly (about 90%) of tubulin subunits, with the remaining 10% consisting of the microtubule-associated proteins (MAPs) that, according to the order of its electrophoretic mobility, were classified as MAP1, MAP2, and tau [2]. Later on, improved electrophoretic techniques allowed further fractionation of MAP1 into MAP1A, MAP1B, and MAP1C (a dynein subunit) [3]. Moreover, different isotypes were described for MAP2 and tau proteins [2]. Tau protein was first isolated at Kirschner’s lab in 1975 [4] and from that year up to 1988, the study of tau shifted from that of a microtubule-associated protein [5,6,7,8] to that of a component of the paired helical filaments found in the brain of Alzheimer’s disease (AD) patients [9,10,11,12,13,14,15,16,17,18]. To date, the analysis of tau protein has been mainly focused on its dysfunction. Here we review and discuss recent findings about the role of both function and dysfunction of tau protein.

## 2. Tau Function and Dysfunction

### 2.1. Tau Function

In the central nervous system of mammals, tau protein is composed of six different isotypes produced by alternative splicing mechanisms. Three of these isotypes contain three copies of the imperfect 31 amino-acid repeats that constitute the microtubule-binding domain (tau 3R) whereas the other three isotypes contain four repeats (tau 4R) [19]. *In vivo*, the most well-known function for tau is microtubule stabilization [20,21]. Hence, it is difficult to obtain non-neuronal proliferating cells that are expressing tau in a stable form since the presence of tau at high levels makes it difficult to depolymerize interphase microtubules to allow the onset of mitosis [22]. For that reason tau 3R (a weaker microtubule stabilizer than tau 4R) can be more readily expressed at high levels than tau 4R in non-neuronal proliferating cells [23]. Thus, tau is mainly present in neuronal, non-proliferating, differentiated cells [19].

Microtubule stabilization could be the mechanism underlying the role of tau on the development of axonogenesis although, in this process, tau can also play an additional role on the localization of some microtubule plus-end tracking proteins (+TIPs), like end-binding (EB) proteins, at the later stages of axon development [24]. Embryonic hippocampal cultures from tau-deficient mice show an abnormal pattern of axonal growth and a significant delay in maturation; an effect that can be rescued by mating those mice with transgenics overexpressing human tau protein [25].

Interestingly, loss of tau could also result in iron accumulation in neurons. It has been reported that amyloid precursor protein (APP) displays a ferroxidase activity that, coupled with a ferroportin, allows for iron export. Since tau facilitates the traffic of the amyloid precursor protein (APP) to the cell surface, loss of tau leads to iron accumulation in primary neuronal cultures [26].

Regulation of tau function is predominantly achieved through post-translational modifications, primarily phosphorylation at many sites (see [27] for a review). Thus, an increase in tau phosphorylation reduces its affinity for microtubules, resulting in neuronal cytoskeleton instability. Moreover, a gradient of tau concentration exists along the axon in mature neurons, with higher levels at the synapse where it can block the binding of motor proteins and favor the local release of their cargo. Additonally, tau phosphorylation by glycogen synthase kinase-3 (GSK3) at specific residues appears to modulate long-term depression (LTD) [28].

It has been demonstrated that tau is also extensively post-translationally modified by lysine acetylation, leading to impaired tau function and promoting pathological aggregation [29], as discussed below. Intriguingly, it has been recently reported that mammalian tau proteins possess intrinsic enzymatic acetyltransferase activity capable of catalyzing self-acetylation at lysines by using catalytic cysteine residues located at the microtubule-binding domain [30]. It remains to be known whether other possible substrates for this activity may exist.

On the other hand, a possible role for tau in sleep regulation has been proposed [26,31]. The activity of neocortical pyramidal cells during various arousal states was measured in a mouse model of tauopathy, showing that membrane potential oscillations were slower during slow-wave sleep and under anesthesia [31]. The observed changes in activity patterns are due to longer down states and state transitions of membrane potentials.

However, probably the most interesting function for tau protein is its role on long-term depression (LTD). Various persistent modifications in neuronal and synaptic functioning provide the biological basis of learning and memory in neuronal circuits and, among these, long-term synaptic plasticity is thought to play a primary role. Long-term synaptic plasticity appears in various forms of potentiation (LTP) and depression (LTD). LTP is an activity-dependent increase in synaptic transmission/strength between two neurons, whereas LTD is an activity-dependent decrease in synaptic transmission/strength. This reduction in the synaptic strength may facilitate a further loss of the synapse. It has been described that loss of tau prevents LTD and it may result in a deficit in spatial reversal learning [28]. Thus, this role of tau in LTD may lead to impairment of stabilized memories and new learning.

Furthermore, within the MAPT gene there is a cryptic protein, saithoin, located in the intron 9, between exons 9 and 10, [5,32]. This protein may have an antioxidant role, a function that will be lost in the absence of the tau gene.

### 2.2. Tau Dysfunction

Although loss of tau in mice results in some deficits, these are not sufficient to affect the viability of the animal [25,33]. Thus, tau loss of function, except for its role in LTD, may not be the primary cause of the group of pathologies known as tauopathies [19].

There seems to be a general agreement that tauopathies are the consequence of a gain of toxic function due to an increase in the amount of tau protein or its modification [19]. An increase in tau levels has been shown to exist in the most prevalent tauopathy, Alzheimer’s disease (AD) [34,35]. Additionally, there are several post-translational modifications of tau protein, such as phosphorylation, acetylation, glycation, truncation, or glycosylation that could confer a toxic gain of toxic function [19]. In particular, tau hyperphosphorylation seems to lead to toxicity [19]. A recent study of mutant tau transgenic mice has demonstrated that hyperphosphorylated, aggregated tau directly harms proteasomal function *in vivo* [36], although the precise molecular mechanism remains unclear.

The original observation relating to tau pathology and dysfunction was its self-aggregation to form polymers, such as paired helical or straight filaments [6,7,8,9,10,11,12,13,14,15,16,17,18]. There are some tauopathies of familial origin in which tau mutations at specific sites might facilitate its subsequent abnormal aggregation [37]. This self-aggregation takes place mainly through the microtubule-binding regions of the tau protein [5,25]. Hence, it is not surprising that not every tau isotype shows the same capacity for microtubule binding or self-aggregation [38]. The high molecular weight isotype (big tau) present in the peripheral nervous system [39] is an isotype with a lower capacity to self-aggregate. This observation agrees well with the recent report indicating the protective role of the high molecular weight tau isotype present in the longest lived rodent, mouse-sized naked-mole rats [40]. An increase in tau phosphorylation by kinases such as GSK3 has been correlated with increased tau aggregation [41,42]. Recently, it has been suggested that under stress conditions tau can be phosphorylated at threonine 175, inducing GSK3 activation which in turn modifies tau at threonine 231, and leads to pathologic fibril formation [43].

As already mentioned, acetylation of soluble tau has important effects on its properties, including stability, protein-protein interaction, and aggregation. A complex tau acetylation pattern has been recently demonstrated *in vitro* with high-resolution NMR techniques, showing that there are more than 20 acetylated sites within the tau molecules [44].

Moreover, tau acetylation is increased in AD brain lysates, whereas tau acetylation at lysine 174 has been reported to be an early change in AD [45]. Overexpression of a tau mutant mimicking acetylation at that residue in mouse brain led to increased hippocampal atrophy and decreased behavioral performance. Furthermore, treatment of tau transgenic mice with acetyltransferase inhibitors lowered tau acetylation, rescued tau-induced memory deficits, and prevented hippocampal atrophy [45]. All together, these findings highlight tau acetylation as a pathogenic step in AD and tauopathies and open new therapeutic avenues to be explored.

## 3. The Tauopathies and Propagation of Pathology

### 3.1. The Tauopathies

The main risk factor for the most prevalent tauopathy, AD, is aging. Similarly, other tauopathies are also more prevalent above 40 years old. However, several tauopathies have been described at young ages, such as fetal or infantile tauopathies like hemimegalencephaly, tuberous sclerosis complex (TSC), focal cortical dysplasia type 2b, and ganglioglioma [46].

Patients with a developmental disorders, such as Down syndrome (DS), the most common genetic form of intellectual disability [47], have a striking propensity to develop early-onset Alzheimer disease (EOAD), including the accumulation of neurofibrillary tangles (NFT). In spite of several similarities between both pathological processes, DS-specific potential mechanisms for cognitive deficits have been recently proposed, such as an intracellular chloride accumulation mediated by GABA_A_ receptors [48]. In the hippocampus of adult DS mice GABA_A_ seems to be excitatory rather than inhibitory [48]. In the case of AD, it has been proposed that NMDA receptors present at the dendritic spines could favor Aβ toxicity mediated by the presence of the complex Fyn-tau [49]. It will be of interest to know whether tau could play a similar function in the GABA_A_ receptors-containing postsynaptic density in DS.

Pre-senile tauopathies include types of early-onset dementia, such as fronto-temporal dementia (FTD) or familial AD (FAD), whereas sporadic AD (SAD) is the most prevalent advanced-age, senile tauopathy [19]. While rare mutations in the *MAPT* gene lead to FTDP-17-tau, the vast majority of tauopathies is sporadic, non-inherited, with aggregation of the wild-type protein [50]. Curiously, one of these tau mutations could also led to progressive apraxia of speech [51], whereas a single nucleotide polymorphism in *MAPT* has also been identified as an important risk factor for Parkinson’s disease (PD) [52,53].

During the progression of AD pathology, neuronal death leads to intracellular tau being released to the extracellular space. It has been suggested that once tau is in the extracellular space it could become toxic for the surrounding neurons [54]. However, tau transmission from cell to cell could occur by exocytosis and endocytosis without the need for neuronal death to release extracellular tau [54,55,56]. On the other hand, to explain that tau transmission only occurs during neurodegenerative processes and not in normal physiological conditions, it has been proposed that aggregated tau is the toxic form for that spreading [26,57]. However, it is unclear if the endocytosis may take place in any cell type or whether a specific receptor, such as muscarinic receptors, are required [55].

Generally speaking, there are three main characteristics for a tauopathy: (a) an increase in tau levels; (b) a modification, like hyperphosphorylation [58,59], sometimes related to another posttranslational modifications such as truncation [60] or acetylation [45]; and (c) an abnormal tau aggregation [61]. Additionally, in some tauopathies a change in tau 3R/4R ratio could facilitate the onset of the disorder.

Regarding tau levels, it is important to know how human *MAPT* gene expression takes place [62]. The role of different types of tau post-translational modifications has been already mentioned, although focusing on truncation, it should be indicated that toxic tau fragments could arise by truncation at both ends (*N*-terminal or C-terminal) of the tau molecule [63,64]. Moreover, tau truncation could modulate tau spreading [65]. Finally, changes in tau 3R/4R ratio may result in differences in microtubule stability. Recently, microtubule dynamics has been correlated with some neurodegeneration disorders [66]. Some diseases, like AD, could be associated to a decrease in microtubule stability, whereas others, like heredity spastic paraplegia, could be rather linked to the presence of hyper stable microtubules [66]. Tau 3R and tau 4R isoforms result from different alternative splicing events. Interestingly, it has been recently described that Huntington’s disease could also be a tauopathy resulting from an increase in the tau 4R/3R ratio [67].

### 3.2. Propagation of Tau Pathology

In the brains of AD patients, tau pathology propagates following an anatomically-defined pattern, from the entorhinal cortex through the hippocampus and into the limbic and associated cortexes, which correlates with the clinical cognitive status of the patient [68]. A body of evidence has been gathered in recent years that strongly suggests that accumulation of abnormal tau is mediated through spreading of protein seeds from cell to cell and involving extracellular tau species as the main agent in the interneuronal propagation of neurofibrillary lesions and spreading of tau toxicity throughout different brain regions [69,70].

Long regarded primarily as an axonal protein, tau also accumulates in the somatodendritic compartment during AD [71], and mislocation to dendritic spines may lead to synaptic dysfunction [72,73]. The presence of tau in the synapse in healthy brains suggests a role for tau in regulating normal synaptic function, whereas during neurodegeneration tau synaptotoxicity seems to be related to soluble forms rather than insoluble aggregates [74]. Measurement of tau synaptic levels in synaptosomal fractions from human *post mortem* AD brains has shown that tau is normally localized in cortical synaptic terminals and that tau cleavage may facilitate tau aggregation and secretion from the pre-synaptic compartment [75]. Interestingly, a trans-synaptic mechanism of spreading of pathology through anatomically-connected neuronal networks has been recently shown in transgenic animal models of tauopathy [57,76], which supports neuropathological studies in *post mortem* brains from argyrophylic grain disease (AGD) [77].

Remarkably, intracerebral inoculation of synthetic preformed tau fibrils induced NFT-like inclusions that propagated from injected sites to connected brain regions in a time-dependent manner [78]. Furthermore, conformation-specific trans-cellular propagation of tau fibrils after release into the extracellular space and subsequent triggering of aggregation in recipient cells by contacting native protein has been shown in co-culture experiments [79]. Intriguingly, using a lentiviral-mediated rat model, it has been shown that human wild-type tau protein trans-synaptically spreads much faster that mutant tau [80]. More recently, using artificial neuronal circuits *in vitro*, it has been further demonstrated that non-synaptic and synaptic mechanism act in parallel to promote tau spreading [81]. Another exciting recent finding is that microglia depletion drastically suppresses propagation of tau pathology in an AAV-based mouse model [82]. Furthermore, microglia are able to secrete tau via exosomes and inhibiting exosome synthesis significantly diminishes tau propagation *in vivo*.

Taken together, cell-to-cell spreading of abnormal tau and toxicity provides a mechanism for tau-targeted immunotherapies as therapeutic strategy for AD and tauopathies (see below).

## 4. Therapeutic Strategies

### 4.1 Therapeutic Targets

Therapies can be designed to reverse a loss of function or to correct a gain of function. With regard to tau loss of function, since it can result in iron accumulation in some specific neurons, the use of an iron chelator, clioquinol, has been proposed [83]. However, as already mentioned, tauopathies are mainly the consequence of (1) an increased tau protein level; (2) increased post-translational modifications; (3) increased aggregation; or (4) altered tau 3R/4R ratio in some specific neuronal populations.

MicroRNAs (miRNAs) have been linked to neurodegenerative processes and its dysregulation contributes to tau neurotoxicity. The highly-conserved brain miRNA miR-219 has been shown to be decreased in *post mortem* brains from AD and also severe primary age-related tauopathy. miR-219 binds directly to the 3’-UTR of the tau mRNA and post-transcriptionally represses tau synthesis, suggesting that this pathway could be used as a possible therapy [23]. On the other hand, the use of specific tau kinase inhibitors (for example lithium or tideglusib for GSK3 or tamoxifen for cdk5) [84] has been proposed but, at present, no clear results have been obtained in clinical trials [85,86]. Post-translational modifications involving tau cleavage resulting in the appearance of truncated toxic tau fragments have been reported [87] and the use of protease inhibitors has been suggested as potential therapy since the use of an uncleavable tau mutant shows attenuated pathological and behavioral defects in a tau transgenic model [87]. Moreover, several compounds able to inhibit formation of tau oligomers and fibrils have already been tested in different animal models [88,89]. A methylene blue derivative is currently being tested in phase III clinical trials for AD and FTD [90]. Curiously, methylene blue was already used by Cajal to stain dendritic spines [91], a structure that contains tau protein [92]. On the other hand, it has been reported recently that a highly conserved PDZ (an acronym combining the first letters of three proteins: post synaptic density proteins (PSD95), Drosophila disc large tumor suppressor (Dlg1), and zonula occludens-1 protein (zo-1); which were discovered to share the domain) serine protease, HTRA1, is able to untangle and chop up tau fibrils in an ATP-independent fashion, decreasing the aggregate burden in a cellular model of cytoplasmic tau aggregation and suggesting some therapeutic potential [93].

### 4.2. Immunotherapeutic Tau Approaches

Immunotherapy for various neurodegenerative diseases has recently emerged as a promising approach for the clearance of pathological proteins in these disorders. The immunotherapy approach is based on eliciting anti-tau antibodies able to clear tau molecules that negatively affect neuronal viability, thus resulting in clearance of tau pathological species and eventually neuronal function improvement. Newly aggregated intracellular tau that transfers between co-cultured cells can provide a model for tau-targeted immunotherapies for AD and tauopathies [94,95]. Both passive and active immunization approaches have been pursued in recent years and have shown potential in animal models.

Although the active immunization approach has certain advantages, it may have autoimmune side effects that can be avoided with passive immunization. Several passive immunotherapy approaches targeting tau with specific antibodies have also been reported recently [96]. Specific phospho-tau antibodies have been used recently to prevent the induction of tau pathology in both primary neuronal cultures and in animal models of propagation of tau pathology [97] showing a significant decrease in tau spreading after systemic administration. Interestingly, not every phospho-specific tau antibodies shows efficacy at preventing tau pathology in animal models and some of them seem to even exacerbate pathology [98]. It is also worth mentioning the use of antibodies specifically targeting cis conformation in specific tau phospho-epitopes in animal models of traumatic brain injury, preventing tau spreading and pathology development [99,100]. Noticeably, intravenous injection of a tau oligomer-specific monoclonal antibody in aged APP transgenic mice led to the removal of age-dependent tau oligomers, reversed memory deficits and shifted the Aβ pathway towards plaque formation [101], also highlighting a mechanistic interaction between tau oligomers and Aβ.

It has been suggested that the most likely mechanism of action for anti-tau antibodies is targeting tau released from cells [102], although several different mechanisms of antibody clearance of tau are likely to act in concert. Determining and targeting, specifically, the most toxic tau species will definitely increase the therapeutic efficacy.

Some clinical trials of tau immunotherapy are already ongoing [103,104] and several more are likely to be initiated in the near future. The recent development of imaging-based biomarkers [105] will enable the progression of tau pathology to be tracked in living patients and greatly facilitate the early-phase testing of tau immunotherapy and other tau-based therapeutic strategies.

## 5. Conclusions

We have reviewed recent developments in tau biology relevant to AD and tauopathies. It has become increasingly clear that, apart from the well-established intracellular functions of tau in microtubule stabilization and axonal transport, intracellular and extracellular tau have important signaling roles that could contribute to the neurodegenerative process in AD and related tauopathies. In addition, the presence of tau in synaptic regions of healthy brain suggests that tau may play a role in the regulation of normal synaptic function. Furthermore, recent studies have suggested that misfolding of tau in diseased brains leads to abnormal conformations of tau that can be transferred to surrounding neurons. Thus, pathological progression seems to involve transmission of tau protein via a potential prion-like seeding mechanism resulting in neurodegeneration in susceptible brain regions.

Some important questions still need to be clarified, such as selective neuronal vulnerability, the exact nature of the tau species involved, or the precise seeding/templating mechanisms, among others. More research is needed to identify disease mechanisms driving release of tau from neurons and propagation of tau pathology and to determine the impact of extracellular tau on cognitive decline during neurodegeneration. The observation that misfolded tau can be secreted and taken up by adjacent neurons calls for the development of novel strategies to block the propagation of tau pathology in the brain, such as immunotherapies. The next few years will certainly bring new insights into the cellular mechanisms underlying tau secretion and uptake, likely identifying novel therapeutic approaches aimed at interfering early on in the process of propagation of tau pathology.
